# *Chlamydia abortus* in Pregnant Woman with Acute Respiratory Distress Syndrome

**DOI:** 10.3201/eid2603.191417

**Published:** 2020-03

**Authors:** Nicolas Pichon, Laure Guindre, Karine Laroucau, Muriel Cantaloube, Agathe Nallatamby, Simon Parreau

**Affiliations:** Hospital Dubois, Brive La Gaillarde, France (N. Pichon, L. Guindre, M. Cantaloube);; ANSES, Maisons-Alfort, France (K. Laroucau);; University Hospital Dupuytren, Limoges, France (A. Nallatamby, S. Parreau)

**Keywords:** Chlamydia abortus, pregnancy, acute respiratory distress syndrome, vaccine, zoonosis, bacteria, rural health, France

## Abstract

We describe a case of *Chlamydia abortus* in a woman in rural France who was pregnant, developed severe generalized infection, and suffered fetal loss. The case stresses the need for healthcare personnel to perform thorough anamnesis in pregnant women in farming areas and to advise them to avoid contact with small ruminants.

A 27-year-old pregnant woman in week 23 of gestation with no other known medical conditions was admitted to a hospital in rural France for influenza-like illness, headache, dry cough, and fever (38.3°C). Ultrasound examination showed a single viable unaffected fetus and no abnormalities in the placenta or amount of amniotic fluid. Laboratory data revealed a raised C-reactive protein of 159 mg/L (reference <12 mg/L) and pronounced thrombocytopenia (platelet count 27 G/L [reference range 150–350 G/L]) without leukocytopenia. Results of urinalysis and blood cultures, including routine bacterial diagnosis, were negative, as were maternal serologic tests for toxoplasmosis, cytomegalovirus, rubella, and syphilis. Results of serologic tests for parvovirus B19, Epstein-Barr virus, and varicella zoster virus were positive for IgG. PCR results for *Chlamydia trachomatis* were negative from vaginal swab specimens, and results of an amniocentesis were negative. 

Probabilistic treatment was started with cefotaxime and metronidazole, but the patient’s condition did not improve over the next 48 hours. She had arterial hypotension (96/55 mm Hg), acute respiratory distress syndrome (PaO_2_/FiO_2_ 114 mm Hg), and hypercapnia (PaCO_2_ 68 mm Hg). She was admitted to the intensive care unit, where she was intubated and placed on a ventilator. Her chest radiographs were consistent with acute respiratory distress syndrome (Figure, panel A). A follow-up pelvic ultrasound showed death of the fetus in utero, and clinicians conducted a delivery. 

**Figure:**
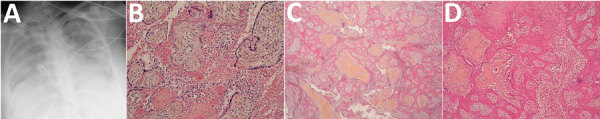
Radiographic and histologic images from a pregnant woman in rural France infected with *Chlamydia abortus*. A) Chest radiograph demonstrating bilateral pulmonary edema consistent with acute respiratory distress syndrome; B) hematoxylin and eosin (H&E) stained section of placenta showing acute histiocytic intervillitis zones (original magnification ×200); C) H&E stained section of placenta showing intervillitis hemorrhage (original magnification ×100); D) H&E stained section of placenta showing necrotizing mural arteritis (original magnification ×100).

When we queried the patient’s husband on her history, we learned he was a goat farmer. He also informed us about an increased number of abortions among the herd during the previous 2 years and that the goats were not vaccinated against *Chlamydia abortus*. Despite absence of direct contact between his wife and the animals, we immediately changed the patient’s antimicrobial drug therapy to doxycycline. The patient improved clinically and biologically and was discharged from the hospital 3 weeks later.

Histopathologic examination of the placenta showed acute villitis and intervillitis on the maternal side (Figure, panels B–D). Immunohistochemistry using antichlamydial lipopolysaccharide antibody and *C. abortus*–specific antibody did not identify chlamydia in the placenta. However, high-resolution melt PCR analysis of isolates from placenta tissue was positive for a wild-type *C. abortus* but not for the live vaccine strain. 

Human infection with *C. abortus* is rare but devastating (*1*). *C. abortus* induces pelvic inflammatory disease in pregnant women and replicates in the trophoblast epithelium, which leads to placental dysfunction and late-term fetal death (*2*–*4*). The infection in pregnant women often requires hospitalization in an intensive care unit and causes fetal death (*5*). Most reported cases of human infection involve direct contact of pregnant women with infected animals, but indirect contact by visiting or living on or close to a farm affected by enzootic abortion also has been described (*6*). When pregnant women, especially those who live in rural areas, arrive in the emergency department with rapidly worsening influenza-like illness, a thorough patient history is necessary, and clinicians should give special attention to possible contact with animals from an infected herd (*5*). In this case, delayed information on the husband’s occupation and the high rate of abortions in the herd explained why zoonotic infection, especially *C. abortus*, was not included in initial diagnostic tests and treatment until chlamydia was excluded. 

The route of transmission to humans for *C. abortus* is uncertain, but direct contact with infected placenta and infective secretions are likely routes. The risk is limited mainly to those actively working with small ruminants, including veterinarians, and their immediate families. Large amounts of *C. abortus* are discharged through vaginal fluids of infected animals <2 weeks before and <2 weeks after abortion. Even without abortion, bacteria are eliminated through the placenta and vaginal fluids during labor in infected animals. Urine, milk, and feces also can contain small amounts of the bacteria following abortion. Women are at risk if they have close contact with small ruminants, but infection also has been associated with handling contaminated clothing and boots. We believe this patient had an indirect mode of transmission.

No effective human chlamydia vaccines are available (*7*). An early, phase 1 clinical trial assessed the safety and immunogenicity of a novel *C. trachomatis* vaccine in humans (*8*,*9*), but that vaccine would not be effective against *C. abortus*. An attenuated *C. abortus* vaccine is available for use in small ruminants but is not safe or approved for use in humans. Developing a human vaccine or adapting the existing veterinary vaccine to humans for such a rare zoonotic infection is not cost-effective. Instead, healthcare personnel should be educated on the importance of thorough anamnesis, and pregnant women living in farming areas should be informed about zoonotic infections. Prevention is the best course against human infection with *C. abortus*, but early recognition, targeted laboratory diagnosis, and appropriate treatment can reduce miscarriage and other effects in pregnant women.
